# Nanoformulation of a Novel Pyrano[2,3-c] Pyrazole Heterocyclic Compound AMDPC Exhibits Anti-Cancer Activity via Blocking the Cell Cycle through a *P53*-Independent Pathway

**DOI:** 10.3390/molecules24030624

**Published:** 2019-02-11

**Authors:** Xuanrong Sun, Longchao Zhang, Mengshi Gao, Xiangjie Que, Chenfeng Zhou, Dabu Zhu, Yue Cai

**Affiliations:** Collaborative Innovation Center of Yangtze River Delta Region Green Pharmaceuticals, Zhejiang University of Technology, Hangzhou 310014, China; 18809883089@163.com (L.Z.); igaomengshi@163.com (M.G.); Que_xj@163.com (X.Q.); zcf19871025@126.com (C.Z.); 15757116042@163.com (D.Z.); chinacaiyue@163.com (Y.C.)

**Keywords:** Pyrano[2,3-c]pyrazole derivatives, nanoformulation, inhibition of cancer cell growth, P21-related S phase and G2 phase arrest

## Abstract

Pyrano[2,3-c]pyrazole derivatives have been reported as exerting various biological activities. One compound with potential anti-tumor activity was screened out by MTT assay from series of dihydropyrazopyrazole derivatives we had synthesized before using a one-pot, four-component reaction, and was named as 6-amino-4-(2-hydroxyphenyl)-3-methyl-1,4-dihydropyrano[2,3-c]pyrazole-5-carbonitrile (hereinafter abbreviated as AMDPC). The IC_50_ of AMDPC against Bcap-37 breast cancer cells was 46.52 μg/mL. Then the hydrophobic AMDPC was encapsulated in PEG-PLGA block copolymers, and then self-assembled as polymeric micelle (mPEG-PLGA/AMDPC) to improve both physiochemical and release profiles. The effect of mPEG-PLGA/AMDPC on BCAP-37 cancer cells showed similar anti-tumor effects as AMDPC. Furthermore, the anti-tumor mechanism of mPEG-PLGA/AMDPC was investigated, which can probably be attributed to stimulating the expression of *P21* gene and therefore protein production on BCAP-37 cells, and then blocked the cell cycle through the P53-independent pathway both in S phase and G2 phase. Thus, mPEG-PLGA/AMDPC is a promising therapeutic agent for cancer treatment, and further in vivo studies will be developed.

## 1. Introduction

Malignant cancer is one of the leading causes of death in humans. Due to the risk of cancer to human life and health, it is always an important task for scientists to find efficient and safe anti-cancer drugs. Nowadays, the mainstream drugs for cancer treatment can be divided into alkylating agents, anti-metabolites, anti-tumor antibiotics, hormones, and platinum compounds according to different drug structures, sources, and different mechanisms of action on tumor cells. Because of drug resistance and the side effects, research and development of anti-cancer drugs has never stopped. 

The proliferation of tumor cells is regulated by their cell cycle. Therefore, cell cycle-related genes and proteins are closely linked with the occurrence and development of tumors, and become targets for tumor treatment [1,2,3,4]. Drugs that can impact the cell cycle are expected to have an anti-tumor effect via inhibiting and delaying tumor growth by affecting the cell cycle—for instance, vinca alkaloids and camptothecin, which can induce M and G_2_ phase arrest, respectively [5,6]. 

The dihydropyrano[2,3-c]pyrazole derivatives play an essential role as versatile synthetic construction blocks and pharmacophores. These compounds are reported to have several pharmacological effects, such as anti-cancer [7], anti-inflammatory [8], anti-microbial [9], molluscicidal [10], and analgesic properties [11]. They also act as biodegradable agrochemicals [12]. In addition, pyrano[2,3-c]pyrazole derivatives act as the inhibitor of cell cycle regulator checkpoint kinase1 (Chk1) [13], which facilitates the maintenance of DNA repair and the regulation of cell cycle checkpoints to ensure the integrity and stability of cell genome, and its antitumor activities have been evaluated in recent years [14,15]. 

PEG-PLGA is one of the biodegradable polymers approved by the U.S. Food and Drug Administration (FDA) as a potent drug carrier. This grafted polymer can form self-assembled polymeric micelles in an aqueous solution, with a hydrophobic core as a drug reservoir and a hydrophilic shell to stabilize the structure. PEG-PLGAs are widely used in drug delivery systems because they can result in better solvability and equal bioactivity compared to pure hydrophobic drugs. Therefore, in this study, we form a nanoformulation to cover hydrophobic AMDPC’s low solubility, evaluate its potential anti-tumor effect, and further investigate its anti-tumor molecular mechanism.

## 2. Materials and Methods 

### 2.1. Materials

**Figure 1:**
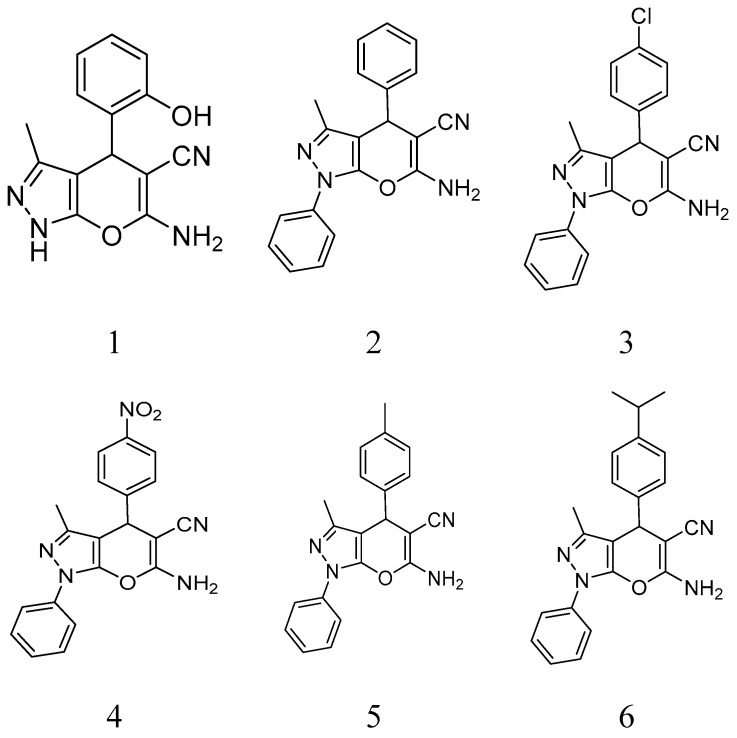

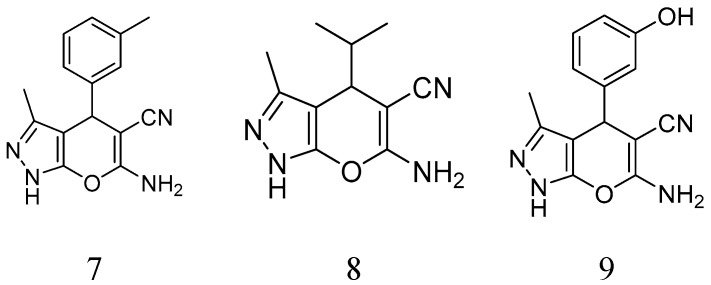
Structure of compounds 1–9.

BCAP-37 and MCF-7 breast cancer cells were generously donated by Dr. Meihua Sui (People’s Hospital of Zhejiang Province); a series of pyrano [2,3-c]pyrazole derivatives were synthesized by Dr. Weike Su’s group (Zhejiang University of Technology) (Figure 1), and have already reported previously [16]. The cell lines were cultivated in RPMI-1640 medium (Gibco, United States), supplemented with 10% fetal bovine serum (Gibco, United States) and antibiotics (10,000 U/mL penicillin and 10μg/mL streptomycin; Gibco, United States). The antibody of P21 protein and goat anti-rabbit IgG were purchased from Abcam, and mPEG-PLGA was purchased from Daigang biological technology company (Shandong, China). Cisplatin was purchased from Sigma Aldrich (St. Louis, MO, USA).

### 2.2. MTT Assay

Cells were seeded at a seeding density of 5000 per well/100 μL medium in a 96-well plate. After 24 h, each well was added with 100 μL fresh medium containing different concentrations of AMDPC (dissolved in dimethyl sulfoxide (DMSO); the final DMSO volume was <0.1%). A medium with 0.1% DMSO was used as a control. After incubated for 24 h, 36 h, and 48 h, 20 μL MTT (5 mg/mL) was added to each well and the plate were incubated for 4 h; then the medium was drained, and 150 μL DMSO was added to each well. Then the color intensity was read at a wavelength of 562 nm. 

### 2.3. Micelle Preparation and Characterization

The procedure for the micelle preparation has been described briefly. A total of 20 mg of mPEG-PLGA and 1 mg AMDPC were dissolved in 4 mL THF and stirred for 1 h. Then the solution was added to 12 mL H_2_O at a flow rate of 1 mL/h gradually, and continuously stirred for 24 h. The solution was dialyzed against water to remove the extra THF and form the micelles. The size and polydispersity of the micelles were evaluated by DLS. The morphology of the micelles was characterized by TEM (Hitachi H7600) following the negative staining of the specimen with 1% uranyl acetate.

### 2.4. Determination of Drug Loading and Encapsulation Efficiency

Lyophilized mPEG-PLGA/AMDPC (5 mg) was dissolved in 2 mL acetone to extract AMDPC for the drug loading and encapsulation estimations. The samples in acetone were first gently shaken on a shaker for 30 min at room temperature, and then evaporated under 35 °C to remove the acetone. The obtained powders were dissolved in 2 mL methanol and filtered through a 220 nm polyester membrane before use. The AMDPC concentration was quantified with a UV-Vis spectrophotometer (Evolution 220, Thermo Scientific, Waltham, MA, United States) at a wavelength of 210 nm. A standard plot of AMDPC (0.5–100 mg/L) was prepared under identical conditions. The AMDPC loading (DL) content and encapsulation efficiency (EE) were calculated as follows:

DL (%) = (Weight of loaded drug/Weight of nanoparticles) × 100%

EE (%) = (Amount of loaded drug/Amount of total drug added) × 100%

### 2.5. In Vitro Drug Release Kinetics 

The lyophilized mPEG-PLGA/AMDPC (20 mg) was dissolved in 3 mL PBS (pH = 7.4) and then transferred to the dialysis bags (MW = 5000) and incubated at 37 °C, shaking gently at 60 r/min. At predetermined time intervals, 0.5 mL of solution was collected, filtered through a 220 nm polyester membrane, and subjected to HPLC analysis to quantify the drug release content (λ_abs_ = 210 nm). 

### 2.6. In Vitro Cytotoxicity of mPEG-PLGA/AMDPC

Standard MTT protocol was followed to evaluate the cytotoxicity of the mPEG-PLGA/AMDPC nanoparticles. Briefly, BCAP-37 cells were seeded in a 96-well plate at 5000 cells per well in 100 μL RPMI-1640 medium, and were allowed to attach overnight. AMDPC nanoparticle solution was added into the well to the designated final concentration and incubated at 37 °C for designate time intervals. Medium with 0.1% DMSO was used as control. A total of 20 μL of 5 mg/mL MTT solution was added to the medium and incubated at 37 °C for 3 h. Then the medium was carefully removed, and the violet crystal was dissolved in 100 μL DMSO and quantified by absorption at λ_abs_ = 562 nm. The relative cell viability (%) was calculated as (OD_562nm_ treated cells – OD_562nm_ blank)/(OD_562nm_no treated cells – OD_562nm_ blank) × 100%.

### 2.7. Quantitative Reverse Transcription Polymerase Chain Reaction

Cells were seeded at a seeding density of 10^5^ per well in a six-well plate. After 24 h, each well was treated with 50 μg/mL mPEG-PLGA/AMDPC for 36 h. The total RNA of cells was extracted with a reagent under manufacturer’s instruction (Gene JET RNA Purification Kit, Thermofisher), and then reverse transcribed with a reverse transcription kit (RevertAid First cDNA Synthesis Kit, Thermofisher). Gene expression was examined using BeyoFastTM SYBR Green qPCR Mix (beyotime, China). The primers are listed in Table S1.

### 2.8. Flow Cytometry Analysis

Cells were seeded at a seeding density of 10^5^ per well in six-well plate. After 24 h, fresh medium was added to each well with mPEG-PLGA/AMDPC at the concentration of 50 μg/mL; then the plate was put into the incubator for 36 h. The distribution of cell cycle was detected using a cell cycle detection kit (beyotime, China). Finally, the stained cells were analyzed by Becton Dickinson FACS flow cytometer (Mansfild, MA, United States).

Flow cytometry was also used to detect levels of protein. Briefly, the cells were fixed with 80% methanol for 15 min, then permeabilized with 0.1% Triton X-100 for 20 min. The cells were incubated with the primary antibody (abcam, Cambridge, MA, United States) for 30 min at 22 °C, followed by the secondary antibody (Alexa Fluor^®^ 488 goat anti-rabbit IgG, abcam, United States) for 30 min at 22 °C. Then the collected cells were detected at the appropriate fluorescence wavelength (E_x_ = 488 nm, E_m_ = 530 nm).

### 2.9. Western Blot Analysis

Cells were treated with 0.1 mg/mL, 0.5 mg/mL, and 1.25 mg/mL mPEG-PLGA/AMDPC (equal to AMDPC’s concentration) respectively for 24 h, then washed with PBS and harvested in RIPA buffer supplemented with a protease inhibitor (1 mM PMSF, 5 μg/mL aprotinin, 5 μg/mL leupeptin) and phosphatase inhibitors (10 mM NaF, 1 mM Na_3_VO_4_). The total cell lysates were subjected to sonication and centrifuged at 14,000× *g* for 15 min at 4 °C. The protein concentration was measured by the BCA protein assay (Thermo Scientific, Rockford, IL) using BSA as a standard. The total proteins (30 or 50 μg) were separated on SDS-PAGE gel, and then transferred to a nitrocellulose membrane (Pall Corporation, Pensacola, FL, United States). The blots were blocked for 1 h at room temperature with 5% skim milk in Tris-buffered saline (TBS) containing 0.05% Tween-20 (TBST). Subsequently, the membranes were incubated with primary antibodies diluted in the 5% nonfat milk TBST solution (1:1000) overnight at 4 °C. After three washes with TBST, the membranes were incubated with horseradish peroxidase-conjugated secondary antibody in the 5% non-fat milk TBST solution (1:5000) for 1 h at room temperature, and washed several times with TBST. The proteins were detected by chemi-luminescence using the ECL Western Blotting Detection Reagent (Amersham Biosciences, Piscataway, NJ, United States) and visualized by a Luminescence analyzer LAS4000 (Fujifilm Medical Systems, Stamford, CT, United States).

## 3. Results

### 3.1. AMDPC Inhibits the Viability of BCAP-37 Cells

**Figure 2:**
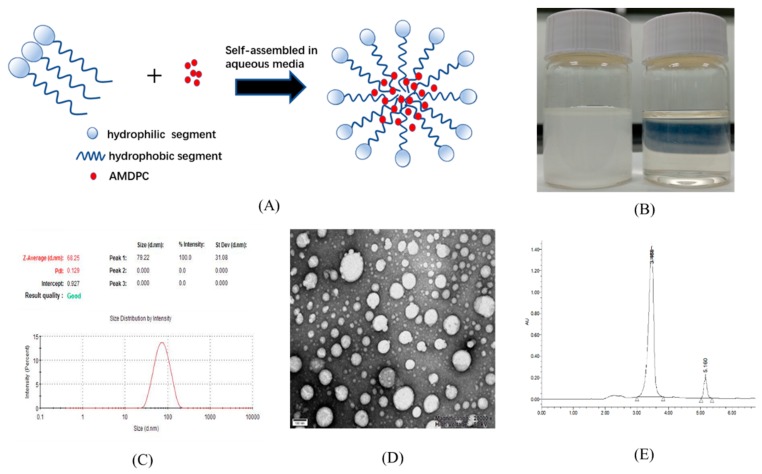
Characterization of 6-amino-4-(2-hydroxyphenyl)-3-methyl-1,4-dihydropyrano[2,3-c]pyrazole-5-carbonitrile (AMDPC)-loaded PEG-PLGA nanoparticles (mPEG-PLGA/AMDPC). (**A**) Schematic diagram for the self-assembled and AMDPC loading mechanism. (**B**) Particle size distribution and dispersion of 50 μg/mL free AMDPC in water (left) and AMDPC-NP (right). (**C**) Dynamic light scattering size measurement of AMDPC-NP. (**D**) Transmission electron micrograph (TEM) image of AMDPC-NP. (**E**) UV-vis absorbance spectra of AMDPC (50 μg/mL).

We evaluated the anti-tumor effect on BCAP-37 and MCF-7 cells via MTT assay of nine pyrano[2,3-c]pyrazole derivatives (Figure 2, Table 1). The results showed that five of the nine compounds have stronger inhibition ability on the BCAP-37 cells than on the MCF-7 cells. Among the nine compounds, the IC_50_ of compound 1 on BCAP-37 cells is 46.52 μg/mL, which is probably the most anti-tumor compound among all. Therefore, we choose compound 1 (6-amino-4- (2-hydroxyphenyl)-3-methyl-1,4-Dihydropyrano[2,3-c]pyrazole-5-carbonitrile) (hereinafter abbreviated as AMDPC) as the object of study, and explored its nanoformulation and mechanism of the effect on the BCAP-37 cells (Table 1).

### 3.2. Characterization of the AMDPC Nanoformulation

Although AMDPC showed potential anti-tumor activity, it was poorly soluble in aqueous solution. Figure 2B (left) shows 50 μg/mL AMDPC at neutral pH. Most of the AMDPC powder was not dissolved, and precipitated at the bottom within a few minutes. To solve this problem, mPEG-PLGA/AMDPC NPs was prepared via the emulsion solvent evaporation method, following typical micellation (illustrated in Figure 2A). The dispersion of AMDPC NPs carrying an equal amount of AMDPC was displayed as a transparent solution of light blue in Figure 2B (right). No precipitation was observed during formulation. The nanoparticles stayed dispersed for several days without visible changes. The size distribution of the polymeric nanoparticles was measured by DLS (Figure 2C). The average particle diameter was 68.16 ± 0.67 nm at 25 ℃ with a narrow size distribution, with PDI equaling 0.139 ± 0.014. The zeta potential of the nanoparticle was −16.87 ± 1.10 mV. TEM analysis revealed a spherically shaped homogeneous nanostructure (Figure 2D).

The entrapped AMDPC contents were estimated by UV-vis spectroscopy (Figure 2E and S1). Acetone was used as a solvent to dissolve the nanoparticles and extract out the AMDPC. AMDPC showed a strong absorbance peak at 210 nm in acetone, while mPEG–PLGA showed no absorbance in the measuring range (Figure 2E). AMDPC was efficiently loaded into the nanoparticles with a DL of 1.28 ± 0.46% (*w*/*w*) and an EE of 64.3 ± 7.89%.

### 3.3. In Vitro Drug Release Kinetics

**Figure 3:**
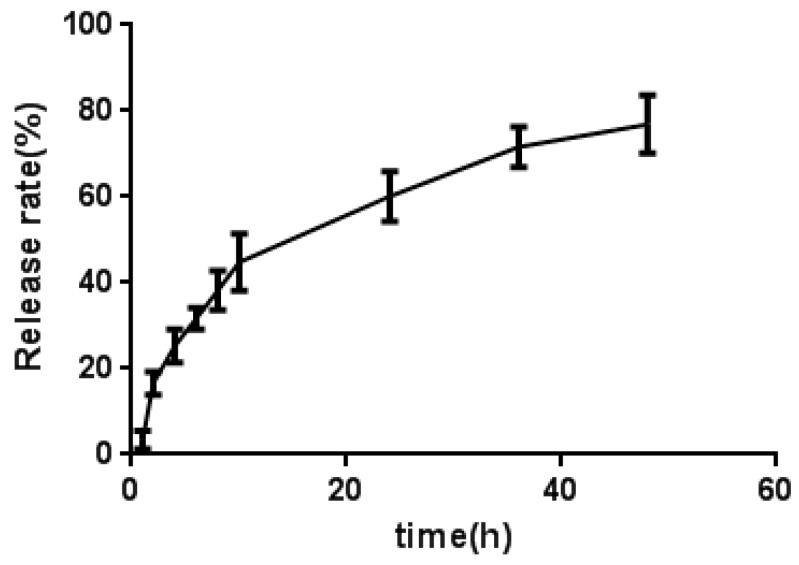
The release curve of mPEG-PLGA/AMDPC in PBS at 37 ℃. Data are presented as mean ± standard error of experiments performed in triplicate.

The in vitro release of AMDPC from mPEG-PLGA/AMDPC into PBS at 37 ℃ is shown in Figure 3. A biphasic release profile was observed: an initial fast release over the first 10 h, followed by a slow and sustained release for a prolonged time period (48 h). After 10 h and 24 h incubation in PBS, 44.8 ± 6.6% and 60.1 ± 5.8% of AMDPC was released from AMDPC-NP, respectively. Overall, about 76.9 ± 6.8% AMDPC was released in 48 h.

### 3.4. In Vitro Cytotoxicity of mPEG-PLGA/AMDPC

**Figure 4:**
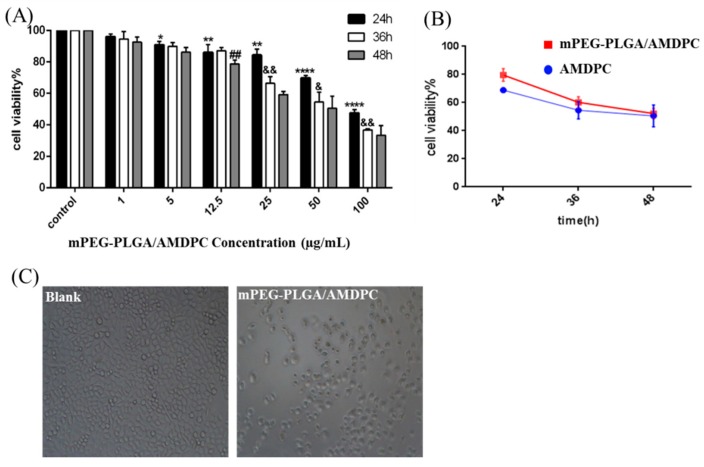
Inhibition ability of mPEG-PLGA/AMDPC on BCAP-37 cells. (**A**) The viability of cells was measured with an MTT assay. The cells were treated with a gradient concentration of AMDPC (0, 1, 5, 12.5, 25, 50, 100 μg/mL) and different time interval. (**B**) Effect of 50 μg/mL AMDPC and mPEG-PLGA/AMDPC on the viability of BCAP-37 cells at different time points. (**C**) The morphology changes of BCAP-37 cells after being treated with 50 μg/mL AMDPC for 36 h. Data represent mean ± SD, (*n* = 3), * *p* < 0.05, ** *p* < 0.01, *** *p* < 0.001, **** *p* < 0.0001 versus control. & *p* < 0.05, && *p* < 0.01 versus 24 h. ## *p* < 0.01 versus 36 h.

The cytotoxicity of mPEG-PLGA/AMDPC was studied in BCAP-37 breast cancer cell lines. As showed in Figure 4A, the viability of the BCAP-37 cells decreased over time and as mPEG-PLGA/AMDPC concentration increased. The higher drug concentration and longer stimulation time can strengthen the inhibition ability against BCAP-37 cells. In the presence of AMDPC (1–100 μg/mL), cell viability has been slightly restrained compared with the control group at 24 h, 36 h, and 48 h. There is no apparent difference between 36 h’ group and 48 h’ group (Figure 4A). Therefore, in the following experiments, 50 μg/mL and 36 h were used as the concentration and stimulation time, respectively, of AMDPC on BCAP-37 cells. 

Furthermore, we found that mPEG-PLGA/AMDPC exhibited almost the same cell cytotoxicity compared with free AMDPC at an equivalent dose about 50μg/mL (Figure 4B). As the incubation time increased from 24 h to 48 h, the viable cells number treated with AMDPC decreased drastically from 70% to 50%; and mPEG-PLGA/AMDPC exhibited the same trend, as the viable cells number decreased from 80% to 52% (*p* > 0.05). In the control studies, cells treated with control-NP did not show any effect on cell viability (Figure S2).

### 3.5. Mechanism of Anti-Cancer Activity on mPEG-PLGA/AMDPC Nanoformulation

#### 3.5.1. Quantitative Reverse Transcription Polymerase Chain Reaction Detection of Gene Expression

**Figure 5:**
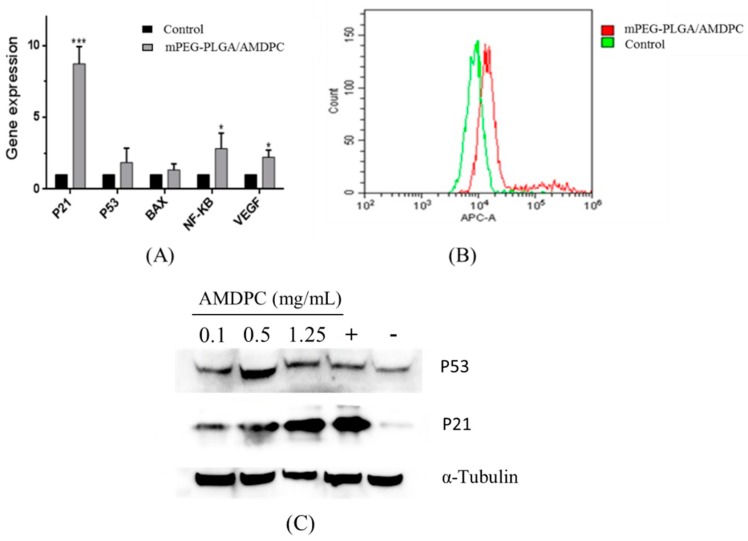
(**A**) The mPEG-PLGA/AMDPC affected gene expression related to the growth and apoptosis of BCAP-37 cells. Cells were treated with 50 μg/mL of AMDPC for 36 h. (**B**) Effect of mPEG-PLGA/AMDPC on P21 proteins of BCAP-37 cells, using flow cytometry analysis. (**C**) Western blots analysis of P21 protein after treatment with mPEG-PLGA/AMDPC on BCAP-37 cells. (**D**) Western blot analysis of P53 protein after treatment with mPEG-PLGA/AMDPC on BCAP-37 cells. The positive control is cisplatin, and the negative control is blank medium. Data represent the mean ± SD (*n* = 3), *** *p* < 0.001, * *p* < 0.05 versus control.

Quantitative reverse transcription polymerase chain reaction (QPCR) results showed that there was a significant increase in the level of *P21*, which has a close connection with cell cycle and growth. The expression level of *P21* was eight times more than in the control group. *P53* and *BAX*, which regulate cell apoptosis, had slight increases compared to the control group. The similar increase was also detected in *NF-κB* and *VEGF*, while the change of both genes was eclipsed by the increase of *P21* (Figure 5A).

Flow results showed that the fluorescence of P21 protein-binding complex had been intensified compared to the control, indicating that mPEG-PLGA/AMDPC could promote the expression of P21 protein (Figure 5B). As shown in Figure 5C,D, P21 protein expression was shown to be up-regulated in a dose-dependent manner, whereas P53 expression was not significantly altered within 36 h’s treatment, compared with the positive control using the clinical anti-cancer agent cisplatin.

#### 3.5.2. Cell Apoptosis and Cell Cycle Assay

**Figure 6:**
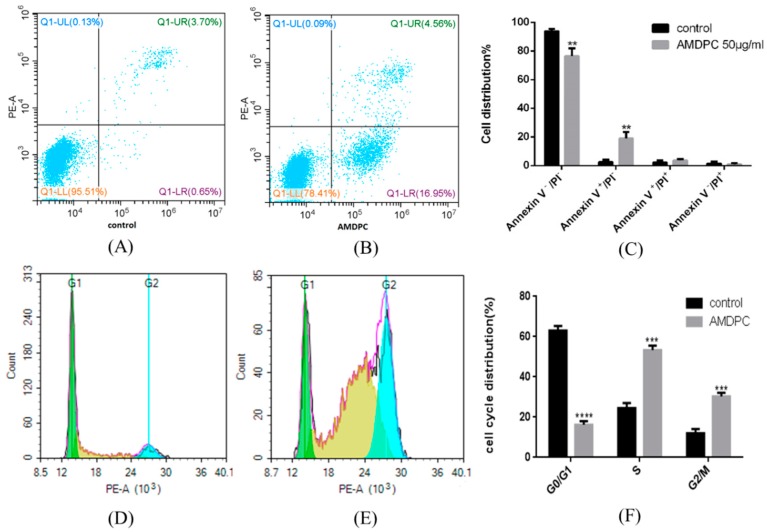
Flow cytometry analysis of cell apoptosis, P21 protein expression, and cell cycle arrest induced by mPEG-PLGA/AMDPC on BCAP-37 cells. Cells were treated with 50 μg/mL of AMDPC for 36 h. (**A**) Control group with no treatments. (**B**) AMDPC-induced BCAP-37 cell apoptosis. (**C**) Cell apoptosis analysis of AMDPC-treated cells. Annexin V and PI were used to stain phosphatidylserine (PS) and nuclei respectively then distinguished the cells from early apoptosis to necrosis by flow cytometry. (**D**) Cell cycle analysis for control group with no treatments. (**E**) AMDPC-induced BCAP-37 cells cycle arrest. Cells were treated with concentration of AMDPC 50 μg/mL for 36 h. Propidium iodide staining was used to determine the DNA content. (**F**) The percentage analysis of BCAP-37 cells in different phases of cell cycle was calculated by flow cytometry. Data represent the mean ± SD (*n* = 3), ** *p* < 0.01, *** *p* < 0.001, **** *p* < 0.0001 versus control group.

Annexin V-PI double staining was performed to detect apoptosis after mPEG-PLGA/AMDPC treatment. The result indicated that compared with control group (Figure 6A), 16.95 ± 4.53% cells were in the Annexin V^+^/PI^−^ zone at the AMDPC equal concentration of 50 μg/mL (*p* < 0.01) (Figure 6C). Most cells were dotted in the Annexin V^−^/PI^−^ zone (Figure 6B). 

A cell cycle assay showed that mPEG-PLGA/AMDPC induced significant changes in cell cycle distribution. The population of cells in S phase and G_2_/M phase had drastically increased by 24.52 ± 2.54%, 12.02 ± 2.12% to 53.38 ± 2.15%, and 30.36 ± 1.82%, respectively, while the proportion of G_1_ phase cells sharply reduced from 63.02 ± 2.27% to 16.26 ± 1.74% (*p* < 0.001) (Figure 6D,F).

## 4. Discussion

This study first screened a series of pyrano[2,3-c]pyrazole derivatives to pursue a potent anti-cancer one, and 6-amino-4- (2-hydroxyphenyl)-3-methyl-1,4-dihydropyrano[2,3-c]pyrazole-5-carbonitrile (AMDPC) had shown strong inhibition ability on BCAP-37 cells among all the compounds. The density of BCAP-37 cells treated with 50 μg/mL AMDPC had sharply decreased, and floating cells in the supernatant did not increase obviously compared with blank group, which indicated that AMDPC may not cause cell apoptosis but may find other ways to play a role.

More than half of all pyrano[2,3-c] pyrazole heterocyclic compound are lipophilic, and their poor water solubility and stability has limited their application in pharmaceutical products [16]. Therefore, strategies are needed to improve the solubility, stability, and in vivo activity. Physical methods, particularly through encapsulation in appropriate delivery systems, have been the most dominant research approach to overcome the limitations of pyrano[2,3-c] pyrazole heterocyclic compounds. Thus, we used PEG-PLGA, which is approved by the FDA as a biodegradable polymer to make a nanoformulation. As illustrated in Figure 2, the nanoparticles were prepared by encapsulating AMDPC in polymer micelles. This formulation resulted in better solvability and equal bioactivity compared to pure AMDPC. Although this nanoformulation requires further characterization in vivo, it is likely to have better stability and activity compared to non-encapsulated AMDPC.

To discover the mechanism of this novel mPEG-PLGA/AMDPC nanoformulation, we first investigated several genes’ expression relating to apoptosis and cell cycle. The expression level of *P21* significantly increased compared to untreated group. However, the expression of *P53*, *Bax*, and *NF-κB*, which had direct connection with cell apoptosis, did not change a lot, which implies that the mechanism of the mPEG-PLGA/AMDPC inhibitory effect is not apoptosis-related, and may be through the other pathways that can upregulate *P21* expressions. The results of flow cytometry and western blot further certified that the level of P21 protein was much higher than the blank group. To further identify whether it was related to a non-apoptosis pathway, we tested the cells’ apoptosis using flow cytometry, and the results showed that only 17% of the cells were quantified at the early stage of apoptosis and treated with mPEG-PLGA/AMDPC at concentration of 50 μg/mL for 36 h, which indicates that the mechanism of anti-cancer effect of AMDPC may not primarily be through the typical apoptosis pathway, but may be through the upregulation of *P21* expression without *P53* participation.

We also investigated the cell cycle after being treated with 50 μg/mL mPEG-PLGA/AMDPC for 36 h. The result indicates that the nanoformulation can block the cell cycle in S and G2 phase. It is speculated that with the treatment, the P21 protein expression is upregulated, and after that the protein could bind to the cyclin–CDK dimer to block the cell cycle in S phase [17]. *P21* is the downstream of the *P53* gene. Since the *P53* expression did not change a lot, *P21* may be activated by other factors such as Sp1/Sp3, Smads and other signal transducers in the absence of a *P53* gene and protein [18]. In conclusion, mPEG-PLGA/AMDPC nanoformulation exhibited an anti-tumor effect through stimulating the expression of the *P21* gene and protein in BCAP-37 cells, thereby blocking the cell cycle in both S phase and G2 phase.

## 5. Conclusions

This study demonstrates a novel pyrano[2,3-c] pyrazole heterocyclic compound AMDPC, which has a potential anti-cancer effect. The mPEG-PLGA/AMDPC compound, which could significantly improve the solubility of AMDPC and make AMDPC release slowly, was prepared later. The mechanism of mPEG-PLGA/AMDPC’s anti-tumor effect on BCAP-37 cells may promote expression of the *P21* gene and protein; the protein induces complete depletion of the G1 phase fraction and cell cycle arrest in S and G2 phase through a *P53*-independent pathway, and therefore, inhibits the cell growth. Therefore, mPEG-PLGA/AMDPC has potential to be a candidate for the development of chemoprevention or therapeutic agents for cancer treatment.

## Figures and Tables

**Table 1:** Half maximal inhibitory concentration (IC_50_) of nine compounds on BCAP-37 and MCF-7 breast tumor cells.

Compound	IC_50_ (μg/mL)	
	BCAP-37	MCF-7
**1**	46.52	>100
**2**	98.62	83.59
**3**	79.17	>100
**4**	67.79	>100
**5**	93.01	>100
**6**	85.56	78.46
**7**	>100	>100
**8**	>100	>100
**9**	88.09	>100

## Supplementary Materials

The following are available online at http://www.mdpi.com/1420-3049/24/3/624/s1, Figure S1: Standard curve of AMDPC, Table S1: QPCR primer sequences (all sequences from 5′ to 3′).

## References

[1] Murray A., Marks D. (2001). Can sequencing shed light on cell cycling?. Nature.

[2] Xiao B., Spencer J., Clements A., Ali-Khan N., Mittnacht S., Broceno C., Burghammer M., Perrakis A., Marmorstein R., Gamblin S. (2003). Crystal structure of the retinoblastoma tumor suppressor protein bound to E2F and the molecular basis of its regulation. Proc. Natl. Acad. Sci. USA.

[3] Akli S., Van C., Bui T., Multani A., Chang S., Johnson D., Tucker S., Keyomarsi K. (2007). Overexpression of the low molecular weight cyclin E in transgenic mice induces metastatic mammary carcinomas through the disruption of the ARF-p53 pathway. Cancer Res..

[4] Bedrosian I., Lu K., Verschraegen C., Keyomarsi K. (2004). Cyclin E deregulation alters the biologic properties of ovarian cancer cells. Oncogene.

[5] Pommier Y., Marchand C. (2011). Interfacial inhibitors: targeting macromolecular complexes. Nat. Rev. Drug Discov..

[6] Li C., Gu Y., Han Y., Zhou K. (2003). Research progress in camptothecin and its analogues. Chin. J. Chem..

[7] Wang J., Liu D., Zheng Z., Shan S., Han X., Srinivasula S.M., Croce C.M., Alnemri E.S., Huang Z. (2000). Structure-based discovery of an organic compound that binds Bcl-2 protein and induces apoptosis of tumor cells. Proc. Natl. Acad. Sci. USA.

[8] Zaki M., Saliman H., Hickal O., Rashad A.E. (2006). Pyrazolopyranopyrimidines as a class of anti-inflammatory agents. Z. Naturforsch C..

[9] Prajapati P., Patel P., Patel S. (2012). Synthesis, Characterization and Antimicrobial activity of 6-amino-4-(substitutedphenyl)-1-(2,4-dinitrophenyl)-3-methyl-1,4-dihydropyrano[2,3-c]pyrazole-5-carbonitrile derivatives. J. Chem. Pharm. Res..

[10] Abdelrazek F., Metz P., Metwally N., El-Mahrouky S. (2006). Synthesis and molluscicidal activity of new cinnoline and pyrano[2,3-c]pyrazole derivatives. Arch. Pharm. (Weinheim).

[11] Kuo S., Huang L., Nakamura H. (1984). Studies on Heterocyclic Compounds. 6. Synthesis and Analgesic and Anti-inflammatory Activities of 3,4-Dimethylpyrano[2,3-c]pyrazol-6-one Derivatives. J. Med. Chem..

[12] Junek H., Aigner H. (1973). Synthesen mit Nitrilen, XXXV. Reaktionen von Tetracyanäthylen mit Heterocyclen. Eur. J. Org. Chem..

[13] Zachos G., Rainey M.D., Gillespie D.A. (2005). Chk1-dependent S-M checkpoint delay in vertebrate cells is linked to maintenance of viable replication structures. Mol. Cell Biol..

[14] Mandha S.R., Siliveri S., Alla M., Bommena V.R., Bommineni M.R. (2012). Balasubramanian S. Eco-friendly synthesis and biological evaluation of substituted pyrano[2,3-*c*]pyrazoles. Bioorg. Med. Chem. Lett..

[15] Adibi H., Hosseinzadeh L., Farhadi S., Ahmadi F.J. (2013). Synthesis and Cytotoxic Evaluation of 6-Amino-4-Aryl-3-Methyl-2,4-Dihydropyrano[2,3-C]Pyrazole-Carbonitrile Derivatives Using Borax With Potential Anticancer Effects. J. Rep. Pharm. Sci..

[16] Zhou C.F., Li J.J., Su W.K. (2016). Morpholine triflate promoted one-pot, four-component synthesis of dihydropyrano[2,3-c]pyrazoles. Chin. Chem. Lett..

[17] Radhakrishnan S., Feliciano C., Najmabadi F., Haegebarth A., Kandel E., Tyner A., Gartel A. (2004). Constitutive expression of E2F-1 leads to P21-dependent cell cycle arrest in S phase of the cell cycle. Oncogene.

[18] Andrei L., Senthil K. (2005). Lost in transcription: P21 repression, mechanisms, and consequences. Cancer Res..

