# Supplementary material for: Nanoformulation of a Novel Pyrano[2,3-c] Pyrazole Heterocyclic Compound AMDPC Exhibits Anti-Cancer Activity via Blocking the Cell Cycle through a P53-Independent Pathway
Source: Molecules. 2019 Feb 11;24(3):624. doi: 10.3390/molecules24030624 (PMC6384735; doi:10.3390/molecules24030624)
Supplement: Supplementary file 1 [file molecules-24-00624-s001.pdf]

Supplementary Materials

Table S1. QPCR primer sequences (all sequences from 5′ to 3′)

Primer	Forward	Reverse
β-actin	TTGCCGACAGGATGCAGAAGGA	AGGTGGACAGCGAGGCCAGGAT
P21	CCTCATCCCGTGTTCTCCTTT	GTACCACCCAGCGGACAAGT
P53	TGTCCTTCCTGGAGCGATCT	CAAACCCCTGGTTTAGCACTTC
Bax	TGCTTCAGGGTTTCATCCAGG	TGGCAAAGTAGAAAAGGGCGA
NF-κB	GGGAAGGAACGCTGTCAGAG	TAGCCTCAGGGTACTCCATCA
VEGF	AGAAGGAGGAGGGCAGAATC	ATCCGCATAATCTGCATGGT

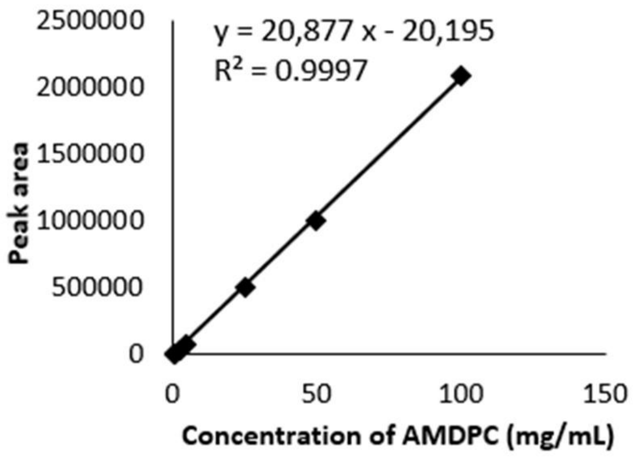

Figure S1. Standard curve of AMDPC

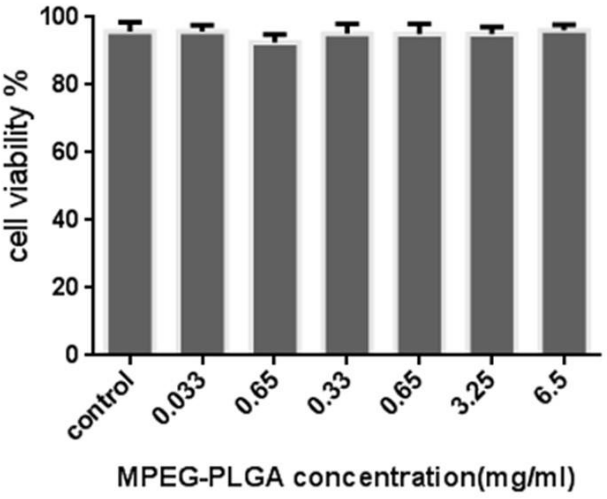

Figure S2. Effects of different concentrations of mPEG-PLGA on viability of BCAP-37 cells for 36h
